# Supplementary figures and images for: Global risk factor analysis of myopia onset in children: A systematic review and meta-analysis
Source: PLoS One. 2023 Sep 20;18(9):e0291470. doi: 10.1371/journal.pone.0291470 (PMC10511087; doi:10.1371/journal.pone.0291470)

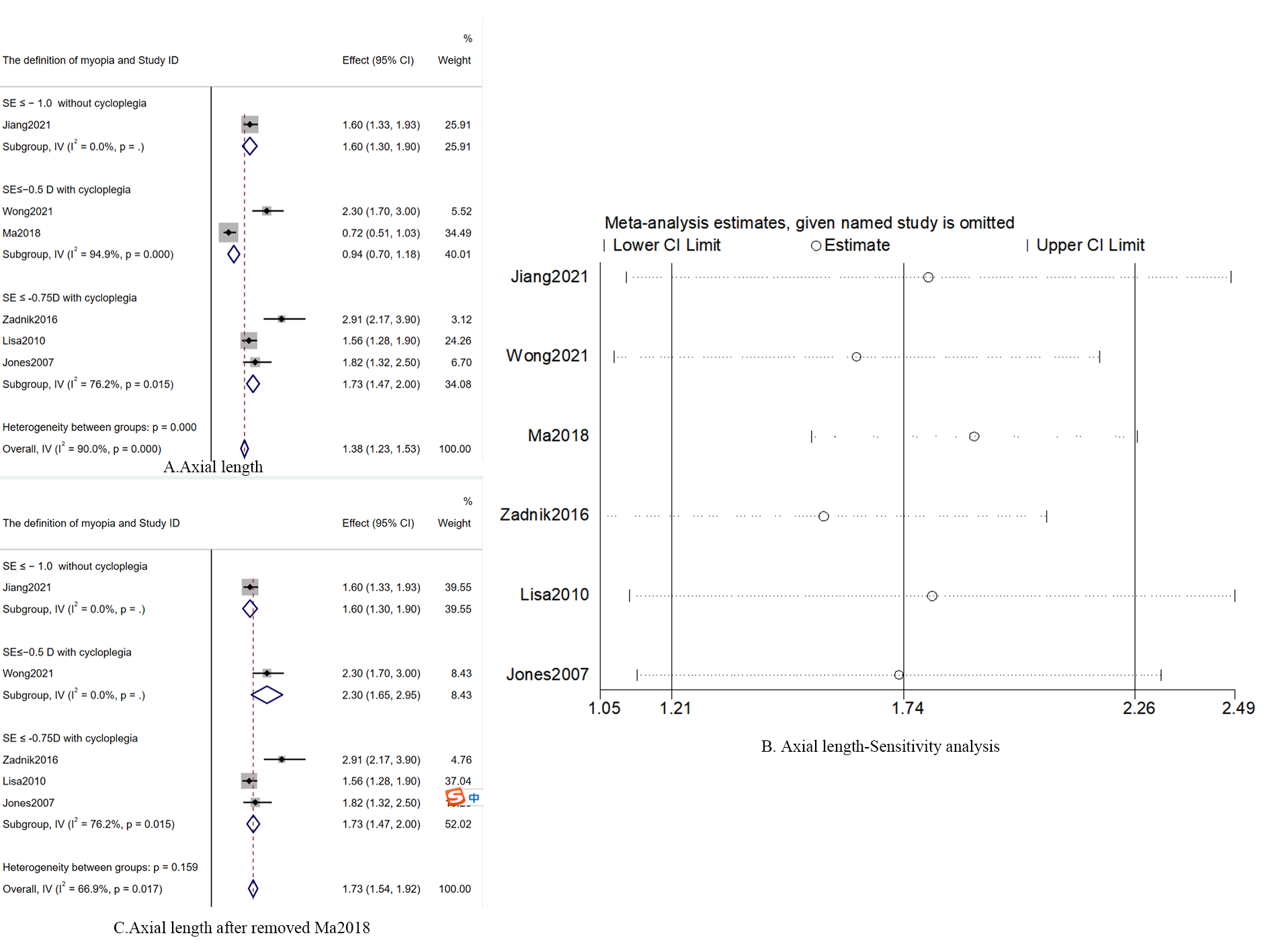

Supplement: S1 Fig — (PNG) [file pone.0291470.s002.png]

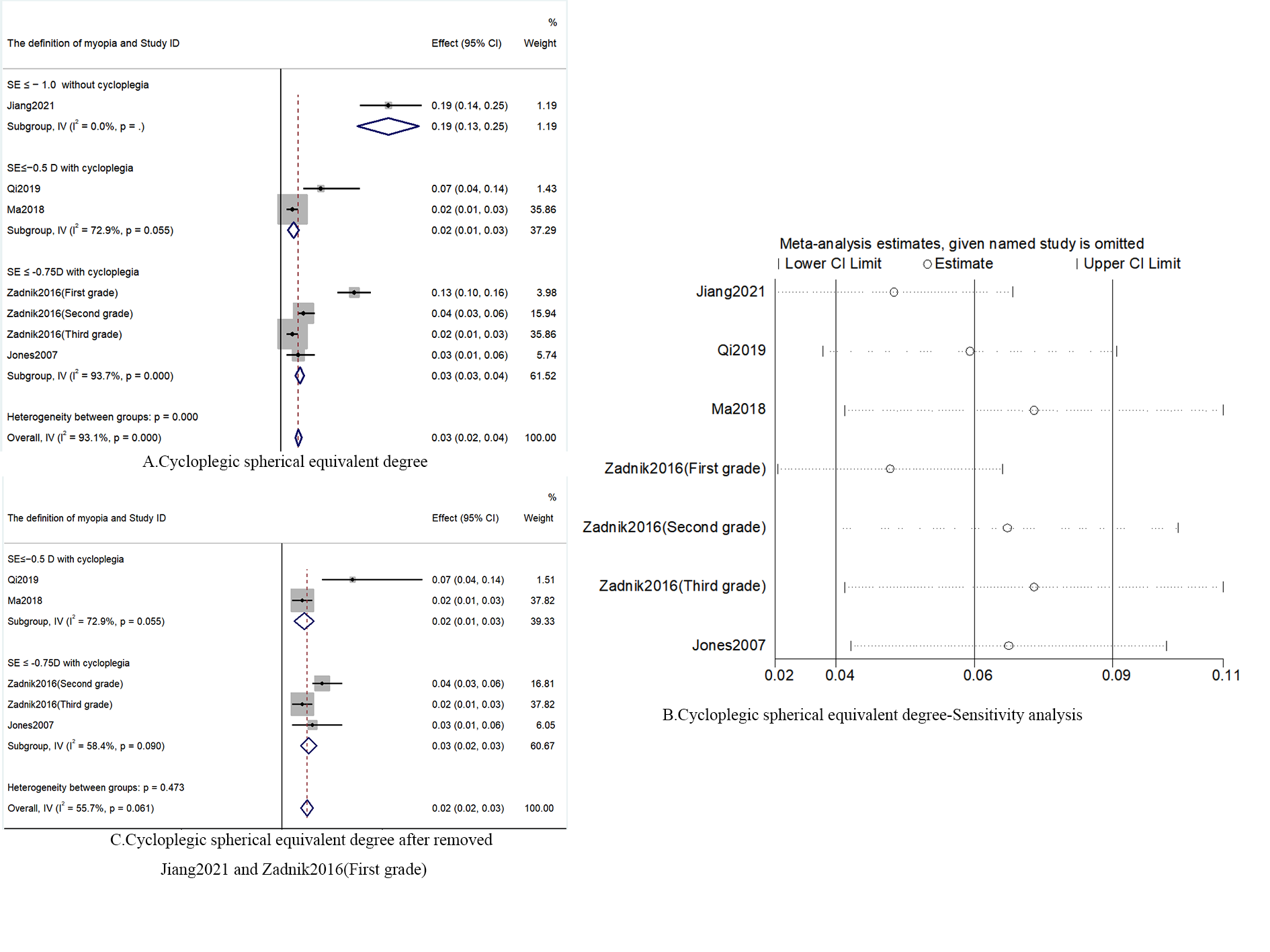

Supplement: S2 Fig — (PNG) [file pone.0291470.s003.png]

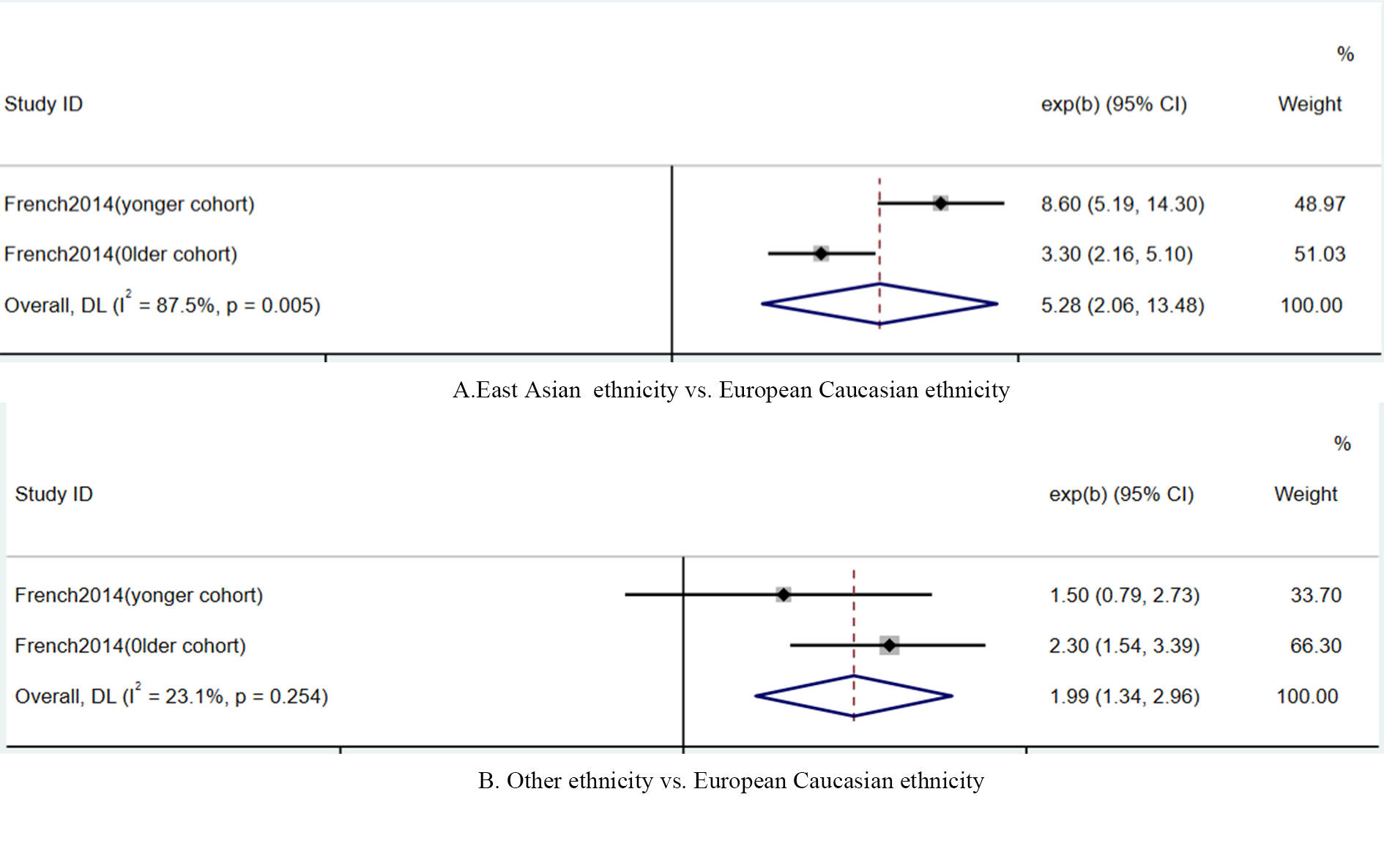

Supplement: S3 Fig — (PNG) [file pone.0291470.s004.png]
