# Supplementary material for: Global risk factor analysis of myopia onset in children: A systematic review and meta-analysis
Source: PLoS One. 2023 Sep 20;18(9):e0291470. doi: 10.1371/journal.pone.0291470 (PMC10511087; doi:10.1371/journal.pone.0291470)
Supplement: S1 Table — (DOCX) [file pone.0291470.s005.docx]

Appendix table 1 The definition and usage of outdoors activities and near work

| **Study ID** | **Outdoors Activities Definition** | **Detail** | **Near Work Definition** | **Detail** |
| --- | --- | --- | --- | --- |
| Li SM2022^[16]^ | Not reported | Not reported | Not reported | Not reported |
| Huang L2021^[17]^ | (1) The frequency of outdoor activity, (2) the duration of outdoor activity, and (3) the overall outdoor factors | (1)≥7 times/week + ≥6 min/time; (2)≥7 times/week + <6 min/time;(3)<7 times/week + ≥6 min/time;(4)<7 times/week + <6 min/time | Watching TV or computers or other fixed electronic screens | Not specify |
| Jiang D2021^[18]^ | Not specify | Low: <1.5 hours/day; Moderate: ≥1.5 hours/day, ≤2.5 hours/day; High: >2.5 hours/day | Watching TV or computers or other fixed electronic screens | Low: 0-2.5 hours/day; Moderate: 2.5-3.5 hours/day; High: >3.5 hours/day |
| Wang BN2021^[19]^ | (1)Light Physical Activity, including slow walking. (2)Moderate Physical Activity, including cycling. (3)Vigorous Physical Activity, including running. | Not specify | Work <50cm(fixed electronic screens, reading), excluding use of TV | Not specify |
| Wong YL2021^[20]^ | Not specify | Not specify | Fixed electronic screens, reading. | Not specify |
| Qi LS2019^[21]^ | Not specify | Low: <9.33 hours; Moderate: ≥9.33 hours, <14 hours; High: ≥14 hours | Not specify | (1)≥28 hours;(2)<28 hours |
| Ma Y2018^[22]^ | Not specify | Low: <4 hours/week; Moderate: ≥4 hours/week,<9 hours/week; High: ≥9 hours/week | Time spent on reading books/magazines and writing homework, watching television, using computer, playing electronic devices such as mobile phone, tablet computer and video games, | Low: <63.5 hours; Moderate: ≥64 hours, <87.5 hours; High: ≥88.0 hours |
| Ma Y2018^[23]^ | Not specify | Not specify | Time spent on reading books/magazines and writing homework, watching television, using computer, playing electronic devices such as mobile phone, tablet computer and video games, | Not specify |
| Wang SK2018^[24]^ | Not specify | Not specify | Watching TV or computers or other fixed electronic screens | Not specify |
| Tsai DC2016^[25]^ | Not specify | (1)<30 min/d;(2)≥30 min/d | Not specify | (1)<2 hours/d;(2)≥2 hours/d |
| Chua SY2016^[26]^ | Not reported | Not reported | Not reported | Not reported |
| Zadnik K2016^[27]^ | Not reported | Not reported | Not reported | Not reported |
| Ma YY2016^[28]^ | Not specify | Low: <4 hours; Moderate: ≥4 hours,<9 hours; High: ≥9 hours | Watching TV | Low: <3.5 hours; Moderate: ≥3.5 hours, <7 hours; High: ≥7 hours |
| Chua SY2015^[29]^ | Physical activities (playing in the backyard, walking or riding a tricycle)  and leisure activities (barbeque, picnic, going to the park or beach) | Not specify | The types of near-work activities included reading or writing, coloring or drawing, playing with handheld devices, and using computers. | Not specify |
| French AN2014^[30]^ | Bike riding, picnics and barbeques, and playing or walking outside; outdoor and near sports; near-based activities, including reading for pleasure; and middistance activities, such as computer use and television viewing. | Younger cohort:low (≤16 hours), moderate (<16 hours,≤23 hours), and high (>23 hours); Older cohort: low (≤13.5 hours), moderate (>13,≤22.5 hours), and high (>22.5 hours) | Watching TV or computers or other fixed electronic screens | Younger cohort:low (≤13 hours), moderate (<13 hours,≤19.5 hours), and high (>19.5 hours); Older cohort: low (≤17 hours), moderate (>17,≤25.5 hours), and high (>25.5 hours) |
| French AN2013^[31]^ | Not reported | Not reported | Not reported | Not reported |
| Jones-Jordan LA2010^[32]^ | Not specify | Not specify | Not reported | Not reported |
| Jones LA2007^[33]^ | Outdoor and/or sports activities | Not specify | Watching television; uses a computer/plays video games | Not specify |
| Saw SM2006^[34]^ | Not reported | Not reported | The types of near-work activities included reading or writing, coloring or drawing, playing with handheld devices, and using computers. | Not specify |
