# Supplementary material for: Global risk factor analysis of myopia onset in children: A systematic review and meta-analysis
Source: PLoS One. 2023 Sep 20;18(9):e0291470. doi: 10.1371/journal.pone.0291470 (PMC10511087; doi:10.1371/journal.pone.0291470)
Supplement: S2 Table — (DOCX) [file pone.0291470.s006.docx]

**Appendix table 2 The risk of bias assessment for each study according to NEWCASTLE-OTTAWA QUALITY ASSESSMENT SCALE**

|  | **Selection** | | | | **Comparability** | **Outcomes** | | |
| --- | --- | --- | --- | --- | --- | --- | --- | --- |
|  | Representativeness of the exposed cohort | Selection of the non- exposed cohort | Ascertainment of exposure | Demonstration that outcome of interest was not present at start of study | Comparability of cohorts on the basis of the design or analysis | Assessment of outcomes | Was follow-up long enough for outcomes to occur | Adequcy of follow up of cohorts |
| Li SM2022^[16]^ | * | * | * | * | ** | * | * | * |
| Huang L2021^[17]^ | * | * | * | * | ** | * | * | * |
| Jiang D2021^[18]^ | * | * | * | * | ** | * | * | * |
| Wang BN2021^[19]^ | * | * | * | * | ** | * | * | * |
| Wong YL2021^[20]^ | * | * | * | * | * | * | * | * |
| Qi LS2019^[21]^ | * | * | * | * | ** | * | * | * |
| Ma Y2018^[22]^ | * | * | * | * | ** | * | * | * |
| Ma Y2018^[23]^ | * | * | * | * | ** | * | * | * |
| Wang SK2018^[24]^ | * | * | * | * | * | * | * |  |
| Tsai DC2016^[25]^ | * | * | * | * | ** | * | * | * |
| Chua SY2016^[26]^ | * | * | * | * | ** | * | * | * |
| Zadnik K2016^[27]^ | * | * | * | * | * | * | * | * |
| Ma YY2016^[28]^ | * | * | * | * | * | * | * | * |
| Chua SY2015^[29]^ | * |  |  | * | * | * | * | * |
| French AN2014^[30]^ | * | * | * | * | * | * | * | * |
| French AN2013^[31]^ | * | * | * | * | * | * | * | * |
| Jones-Jordan LA2010^[32]^ | * |  | * | * | * | * | * |  |
| Jones LA2007^[33]^ | * | * | * | * | * |  | * | * |
| Saw SM2006^[34]^ | * | * | * | * | ** | * | * | * |

Note: A study can be awarded a maximum of one star for each numbered item within the Selection and Exposure categories. A maximum of two stars can be given for Comparability.
