# Supplementary material for: Global risk factor analysis of myopia onset in children: A systematic review and meta-analysis
Source: PLoS One. 2023 Sep 20;18(9):e0291470. doi: 10.1371/journal.pone.0291470 (PMC10511087; doi:10.1371/journal.pone.0291470)
Supplement: S1 File — (DOCX) [file pone.0291470.s007.docx]

### Search strategies

**PUBMED AND Cochrane**

#1 (((((((((((((((myopi*[Title/Abstract]) OR (myopia[MeSH Terms])) OR (shortsight[Title/Abstract])) OR (short-sighted[Title/Abstract])) OR (short-sightedness[Title/Abstract])) OR (short sight[Title/Abstract])) OR (short sighted[Title/Abstract])) OR (short sightedness[Title/Abstract])) OR (near-sight[Title/Abstract])) OR (near-sighted[Title/Abstract])) OR (near-sightedness[Title/Abstract])) OR (near sight[Title/Abstract])) OR (near sighted[Title/Abstract])) OR (near sightedness[Title/Abstract])) OR (refractive errors-[MeSH Terms])) OR (refract*[Title/Abstract])

#2 ((((cohort studies [Title/Abstract]) OR (cohort study [Title/Abstract]))

#3 #1 AND #2

**EMbase**

#1 'degenerative myopia'/exp/MJ OR 'myopia'/exp/MJ OR 'high myopia'/exp/MJ OR 'refraction error':af OR myopi*:ui OR 'short sight*':ui OR nearsight*:ui OR refracti*:ui

#2 'cohort study':ti,ab,kw OR 'cohort studies':ti,ab,kw

#3 #1 AND #2

CNKI **AND VIP AND Wanfang**

#1 Dui lie yan jiu

#2 Jin shi

#3 #1 AND #2
