# Supplementary material for: Global risk factor analysis of myopia onset in children: A systematic review and meta-analysis
Source: PLoS One. 2023 Sep 20;18(9):e0291470. doi: 10.1371/journal.pone.0291470 (PMC10511087; doi:10.1371/journal.pone.0291470)
Supplement: Attachment — Submitted filename: Response to reviewers comments.docx [file pone.0291470.s009.docx]

### View Letter

| **Date:** | Apr 19 2023 11:50AM |
| --- | --- |
| **To:** | "Hongsheng Bi" hongshengbi@126.com |
| **From:** | "PLOS ONE" plosone@plos.org |
| **Subject:** | PLOS ONE Decision: PONE-D-23-06028 |
| **Attachment(s):** | [Review Comments.docx](https://www.editorialmanager.com/pone/download.aspx?guid=%7bbe20319b-b02a-4991-a9f2-9709a499f678%7d&scheme=13&id=496016) |

5. Review Comments to the Author

Reviewer #1: Title: Global risk factor analysis of myopia onset in children: A systematic review and meta-analysis

Manuscript Number: PONE-D-23-06028

The authors conducted a meta-analysis to evaluate the risk factors that may be related to the onset of myopia. This topic is of clinical importance. To provide more objective results, some modification is needed. Here are some suggestions:
Major:
1. In the Introduction section, the authors mentioned that no meta-analyses have been performed to provide a comprehensive overview of risk factors such as age, sex, and household income.
“no meta-analyses have been performed to provide a comprehensive overview of risk factors such as age, sex, and household income” in the introduction section has been deleted. Meanwhile, the content " There are some differences in the risk factors of myopia in children in different regions, so intervention and management should be carried out according to local conditions.” This study provides valuable information for further understanding of the pathogenesis of myopia in children, which can help to develop more precise prevention and treatment measures to promote children's eye health and overall development."

2. Did the authors take urbanization into consideration? Some studies had demonstrated the rural-urban differences in childhood myopia.
In section 4.1 of the article, "The burden of myopia in poor countries is higher than that in developed countries in terms of geographical factors. This shows that we need to strengthen support and assistance for children's vision protection in poor areas." This also indicates that there is a difference between rural and urban vision factors.

3. I suggested that the Results should express in different paragraphs with heading according to factors considered such as heredity factors, personal factors, environment factors, behavior factors and etc..
In section 3.3, subheadings such as genetic factors, gender factors, environmental factors and behavioral factors are added according to your comments, which are expressed in different paragraphs and headings. Make the content of the essay clearer.

4. Only risk of bias graph (Figure 2) was provided, I recommend the authors to provide a figure of risk of bias summary so that each risk of bias item for each included study could be seen.
Bias is the error caused by systematic error, which leads to the deviation of meta-analysis results from the real situation and thus leads to certain misdirection. Risk bias chart is a common financial risk analysis tool designed to help investors find and identify risk bias in portfolios.

5. What does the sentence on page 17 lines 209-211 mean?
Lines 209-211 are the title of Figure 1 and an explanation of what is in the figure. Figure 1 and lower SE27, 30, 32, 36, 42 (RR: 0.03; 95% CI: 0.02 -- 0.04; I^2^: 93.1%; n = 7706. The certainty of the evidence is low; Appendix Figure 2) may be associated with a higher risk of episodes of myopia.

Minor:
1. Consistency of numerals should be addressed, such as 3,578 or 6,9612.
Among the 69612 patients, 3578 cases were selected for analysis through data screening and extraction, and 69652 cases were modified to 3578 cases in the abstract.

2. In the Results section, authors should specify the corresponding results more specifically such as Figure 3A, not Figure 3 only.
Modifications were made on the basis of the suggestions made

3. Some typos must be corrected, such as nigh should be corrected as nine.
Sorry for such typing mistake, and the word "near" is modified to “nine” on page 237,240,241.

4. What does ‘c(6-11)’ mean in the column of the study population of Jones-Jordan LA2010[41] in Table 1?
It refers to the age.

5. What does the level of Nine work time ‘<17 ,≤25.5’ mean in Table 2?
It refers to the working time.

6. The image resolution was not so good. It looks a little vague.

The image quality is improved.

Reviewer #2: Though the author tried very hard to find the global risk factor for myopia, there is no new information or interesting issue to solve that intrigue the reader. (For example, meta-analysis can make conclusions of some controversies that clinicians face) The quality of evidence is low and now useful for the clinicians to refer to.

There are contradictions in the writing. For example, on page 13 line 282, the author said sleep duration may not be associated with myopia onset. However, on page 14 line 292, the author said sleep duration remain controversial and lack sufficient cohort studies for confirmation. There should be consistency in the article, so the readers won’t get confused.
The content of line 292 on page 14 of the article has been revised to ensure the consistency of the article content.

I recommend the author provide more information and deep discussion. For example, on page 15 line 329. The high heterogeneity of different studies. The definition of myopia includes cycloplegic refraction, axial length, and biometric ratio. The author can further compare the pros and cons of definitions. Comprehensive and discussion about the results are suggested.

The influence of different factors on approximation is added in Discussion 4.1.

Reviewer #3: This article is comprehensive in assessing risk factors for the development of myopia, but there are numerous errors throughout that need to be corrected.
Q1.Line 52：“myopia parents” is incorrect.
Sorry for confusion. It has been revised as “children were more likely to develop myopia when their parents suffered from myopia.”

Q2.Line 59-60: The references here did not point to an increased incidence of myopia, but merely suggested that the higher prevalence of myopia.
The content of the article has been revised to elaborate the content of the article.

Q3. Lines 64-66：Missing references.
References have been added again, and thanks for your careful work.

Q4. Lines 71,73, 75, etc.: Sherwin et al, Foreman, Xiong et al. among others. Your citations are inconsistent in many ways; please follow a standard form.
The introduction part is summarized and added more abundant content.

Q5. Line 73: Punctuation needs to be fixed. Additionally, there shouldn't be an s in Device.
Line 73: Punctuation has been changed. In addition, “s” in “devices” have been deleted.

Q6. Lines 80-81: Not factually correct. I understand that that multiple meta-analyses of myopia risk factors have been published, as well as an umbrella review of published meta-analyses of myopia risk factors.
eg: Ying ZQ, Li DL, Zheng XY, Zhang XF, Pan CW. Risk factors for myopia among children and adolescents: an umbrella review of published meta-analyses and systematic reviews. Br J Ophthalmol. 2023 Feb 8:bjo-2022-322773. doi: 10.1136/bjo-2022-322773. Epub ahead of print. PMID: 36754586.
The content of lines 68-79 of the article has been revised. The supplementary content is as follows: there are certain differences in the risk factors of myopia in children in different regions, and intervention and management should be carried out according to local conditions. This study provides valuable information for in-depth understanding of the pathogenesis of myopia in children, which can help to develop more accurate prevention and treatment measures to promote the eye health and all-round development of children.

Q7. Lines 167-168: The content is not consistent with the information in Table 1.
The content of this line has been modified

Q8. Row 170: The content is not consistent with the information in Table 1.
The content of this line has been modified

Q9. Line 181: This sentence is difficult to understand. “Twelve cohorts were included from 11 cohort studies” What does this mean?
The content was modified to report the refractive status of the parents (both myopic and non-myopic) in these cohort studies.

Q10. Line 183: The reference is wrong; twelve cohorts only have 11 references.
The content has been modified, and the specific number of references is no longer stated.

Q11. Lines 190-192: The content does not correspond to the information in Figure 3.
In section 3.3, the relevant content has been modified

Q12. Lines 190-192：“middle certainty of evidence” and “the evidence was considered to have low certainty” are contradictory. What is the basis used for determining middle certainty and low certainty?
The description of determinism has been modified. Here, the significance relationship is described in terms of the P-value.

Q13. Lines 196-198: The content does not correspond to Appendix Figure 2 at all. Why are the results of Appendix Figure 2 presented before those of Appendix Figure 1 up to this point? Please double-check the results again (RR value is 0.03?) for the Appendix Figure 2 summary value.
This paragraph only illustrates the description of Figure 2, cutting out what the figure describes.

Q14. Lines 200-202: Figure 4 depicts 11 articles. The content does not correspond to Figure 4. What is the basis of the middle certainty?

Certainty is a stable state of an objective thing before it has undergone qualitative change. The basis of certainty is based on the values presented.

Q15. Lines 203,204: Incorrect wording. the elderly contradicts Age:12
The “elderly” has been corrected into “children”, and I want to say sorry for such mistake.

Q16. Lines 207-209: The results are contradictory. How can an RR of 0.03 be derived from lower SE may be related to the higher risk of myopia onset?
Related contents have been revised according to your comment.

Q17. Lines 214-218：“The results also showed that a deeper AC, a higher AL/CR ratio, thinner lenses, worse presenting visual acuity, lower magnitude of positive relative accommodation may be more likely to develop to myopia. Besides, children with astigmatism, thicker Crystalline lens, and elevated accommodative convergence to accommodation (AC/A ratio) may decrease the risk of myopia onset”. Are these results from this paper? How were the results obtained? What is the basis? Please organize them in a table.
These results are derived from a summary of the data obtained from the references.

Q18.Line 259-260: What did “the indicators of the eye” mean? Were you referring to the parameters of eyes?
Yes, it refers to “the parameters of eyes”.

Q19.Table 2：“≥64.5, <87“and “ ≥4 <9” lacks of standardization, <17, ≤25.5 contradictory to ≤17
“<17” has been corrected as “17≤”.

Q20.It is advised to change the effect size label in the forest plots to RR or OR instead of Effect.
“effect” means a summary of the data.

Q21.Why was the RR summary effect size chosen for this article, although aggregate measures normally employ OR (95% CI) to examine the connection between potential factors and myopia?
OR differs from RR, but is a very good approximation of RR when the disease is less than 5 percent.

Q22. Table 2's description is overly simplistic, and while this paper uses RR as an effect size throughout the text, why does Table 2 use OR?
OR and RR both refer to how many times the risk of disease of the exposed person is compared to the risk of disease of the non-exposed person. But RR is mostly used in cohort studies. OR is used in case-control studies, where incidence cannot be calculated, and therefore relative risk cannot be calculated. OR can only be used as an indicator of the strength of the reaction association. OR differs from RR for different prevalence and morbidity conditions, but is a very good approximation for RR when the disease is less than 5 percent.

Q23. Lines 70-79. There are numerous examples from other literature that I believe should be included in the discussion rather than the introduction. Furthermore, the introduction is far too brief.

The introduction adds the content. The references in the introduction are a summary of the current research, and on this basis, the purpose of this study is introduced.
