# Supplementary material for: Global risk factor analysis of myopia onset in children: A systematic review and meta-analysis
Source: PLoS One. 2023 Sep 20;18(9):e0291470. doi: 10.1371/journal.pone.0291470 (PMC10511087; doi:10.1371/journal.pone.0291470)
Supplement: Attachment — Submitted filename: Response to Reviewers.docx [file pone.0291470.s010.docx]

PONE-D-23-13955R1
Global risk factor analysis of myopia onset in children: A systematic review and meta-analysis
Mr. Hongsheng Bi

Dear Dr. Bi,

We've checked your submission and before we can proceed, we need you to address the following issues:

1. Please include a figure label and title for Figure 1, 2, 3, 4 and 5 in your main manuscript.
Thanks to the editor's valuable advice, the picture name has been added and put into the article.
2. Please include your tables as part of your main manuscript and remove the individual files. Please note that supplementary tables (should remain/ be uploaded) as separate "Supporting Information" files
Thanks to the editor's valuable comments, the table has been put into the article
3. Please note that funding information should not appear in the Acknowledgments section or other areas of your manuscript. We will only publish funding information present in the Funding Statement section of the online submission form. Please remove any funding-related text from the manuscript.
Thanks to the valuable comments of the editor, the financial information involved in the article has been deleted.
We've returned your manuscript to your account. Please resolve these issues and resubmit your manuscript within 21 days. If you need more time, please email the journal office at plosone@plos.org. We are happy to grant extensions of up to one month past this due date. If we do not hear from you within 21 days, we will withdraw your manuscript.

Please log on to PLOS Editorial Manager at https://www.editorialmanager.com/pone/ to access your manuscript. You will find your manuscript in the 'Submissions Sent Back to Author' link under the New Submissions menu. Be sure to remove your previous manuscript file if you are uploading a new file in response to these requests. After you've made the changes requested above, please be sure to view and approve the revised PDF after rebuilding the PDF to complete the resubmission process.

We are requesting these changes to comply with the PLOS ONE submission guidelines (https://journals.plos.org/plosone/s/submission-guidelines). Please note that we won't send your manuscript for review until you have resolved the above requests.

Thank you for submitting your work to PLOS ONE and supporting our mission of Open Science.


Kind regards,

Maidelyn R. Peregrin
PLOS ONE
